# Supplementary material for: Electro-assisted printing of soft hydrogels via controlled electrochemical reactions
Source: Nat Commun. 2022 Mar 15;13:1353. doi: 10.1038/s41467-022-29037-6 (PMC8924165; doi:10.1038/s41467-022-29037-6)
Supplement: Supplementary file 1 — Supplementary Information [file 41467_2022_29037_MOESM1_ESM.pdf]

# Electro-assisted Printing of Soft Hydrogels via Controlled Electrochemical Reactions


## Author Information

### Affiliations

**1. Department of Automatic Control and Systems Engineering, Faculty of Engineering, University of Sheffield, Sheffield, UK**

**2. Leibniz Institute of Polymer Research Dresden, Dresden, Germany**

Aruã Clayton Da Silva[1], Junzhi Wang[1] & Ivan Rusev Minev [1, 2 *]

### Corresponding author

*Correspondence to: *i.minev@sheffield.ac.uk*

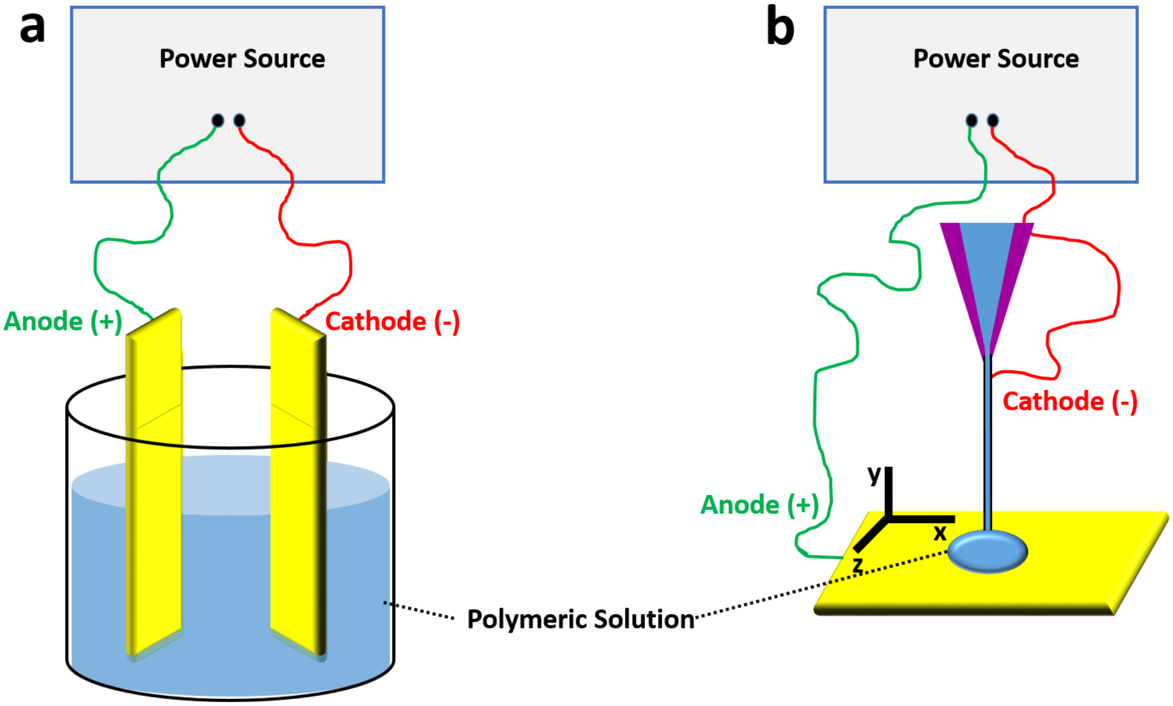


**Supplementary Figure 1 | 2-electrodes configuration system. a** The 2-electrodes configuration with cathode (red) and anode (green) is feasible to be applied using a power source using two gold plates or **b** a gold plate and needle system.

**a** $2\,Cl^-_{(aq)} \rightleftharpoons Cl_{2(g)} + 2\,e^- \qquad E^0 = +1.396V$

**b** $2\,H_2O_{(l)} \rightleftharpoons O_{2(g)} + 4\,H^+ + 2\,e^- \qquad E^0 = +1.230V$

**c** $Au_{(s)} + 4\,Cl^-_{(aq)} \rightleftharpoons AuCl^-_{4(aq)} + 3\,e^- \qquad E^0 = +1.002V$

**Supplementary Figure 2 | Standard potential of electrochemical reactions. a** Chloride ions oxidation reaction to chlorine gas. **b** Water hydrolysis oxidation reaction to oxygen and protons. **c** Gold oxidation in presence of chloride ions to tetrachloroaurate. The standard electric potentials in aqueous solution was extracted from books[1,2]. It is widely known the water electrolysis in presence of chloride can generate hypochlorite species for electrochemical oxidative treatment of organic waste, but it require high electric potentials[3,4]. Considering gold surface, it was reported only tetrachloroaurate and gold oxide (in form of AuO or AuOH) formation on gold electrodes in aqueous media containing chloride ions (and absence, using perchlorate)[5,6]. Additionally, gold is oxidizing to gold(III) (stabilized by complexation with chloride ions) at the electric potential where the deposition of chitosan hydrogel occurs and tetrachloroaurate ion is well known as oxidizing agent[7–9].

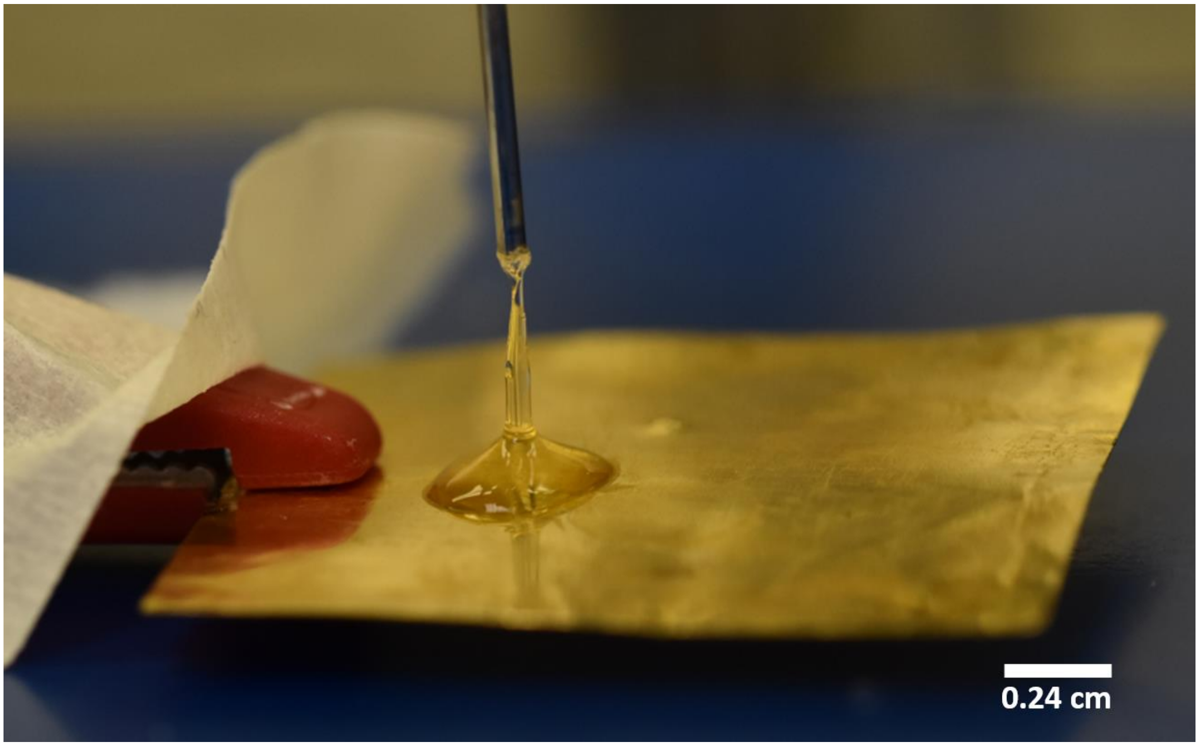


**Supplementary Figure 3 | Precipitated chitosan.** Picture of the precipitated hydrogel of chitosan in the CE (needle) when higher than +2V is applied to the WE.

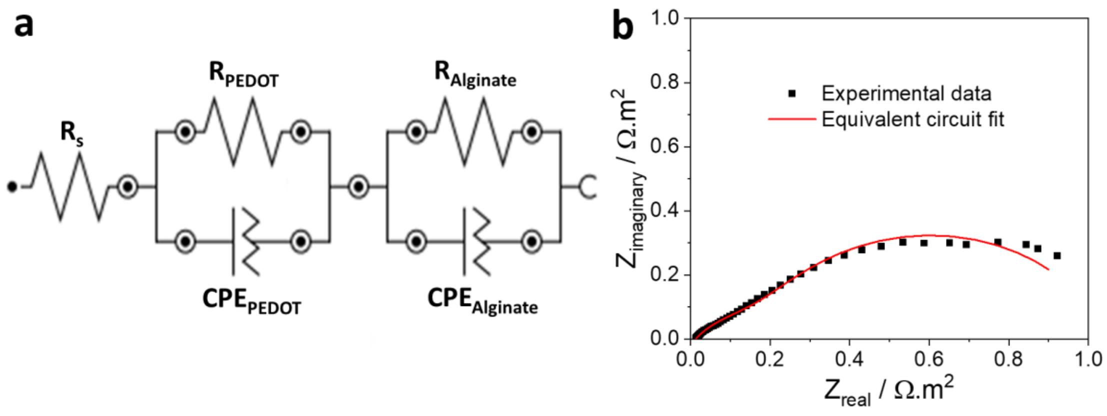


**Supplementary Figure 4 | Fit and simulation for hybrid PEDOT/alginate. a** Equivalent electrochemical circuit for the hybrid PEDOT/alginate. **b** Nyquist plot of the experimental data (black squares) and equivalent circuit fit (red line). The hybrid PEDOT/alginate shows a small semi-circle at low frequencies (PEDOT contribution) and a bigger at high frequencies (alginate contribution). $\chi^2$ for the simulated data was 0.07307.

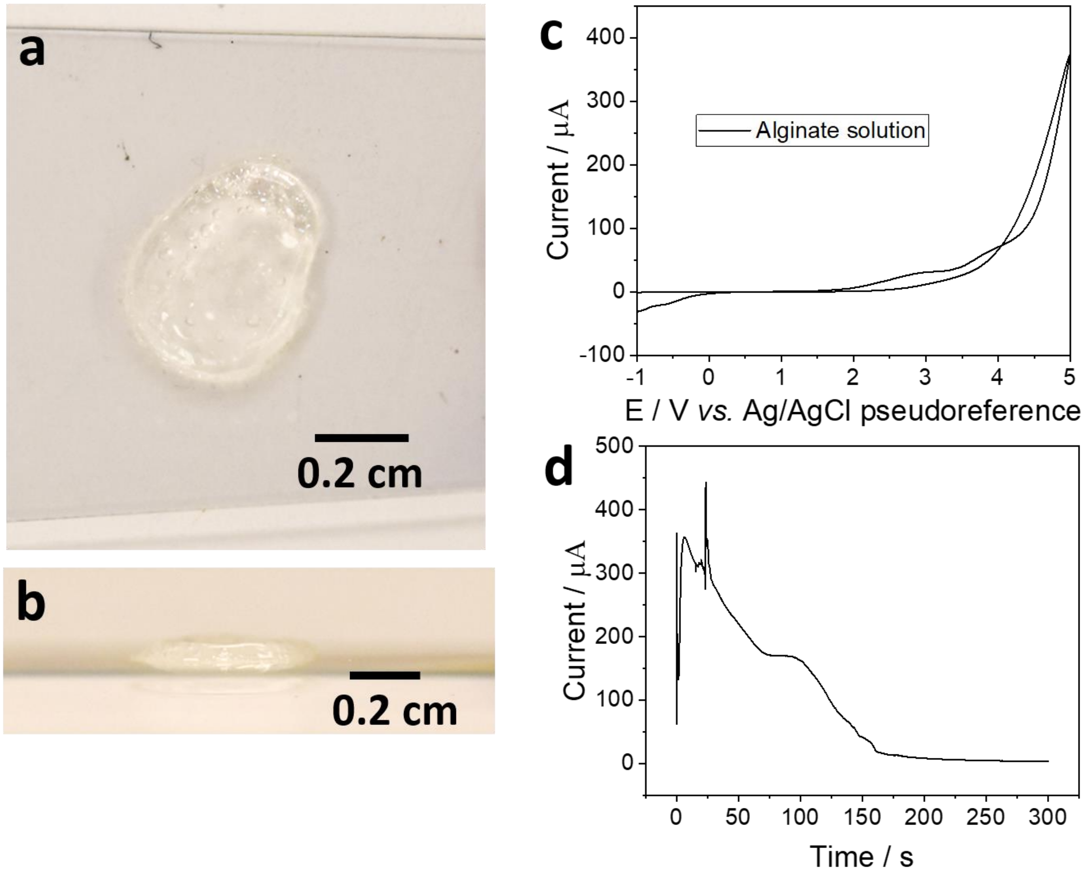


**Supplementary Figure 5 | Alginate hydrogel over ITO/PET substrate. a** Picture of the alginate hydrogel formed in droplet over ITO/PET substrate top view and **b** side view. **c** Cyclic voltammetry of the alginate solution over ITO/PET substrate. **d** Chronoamperometry at +5V for 300 seconds.

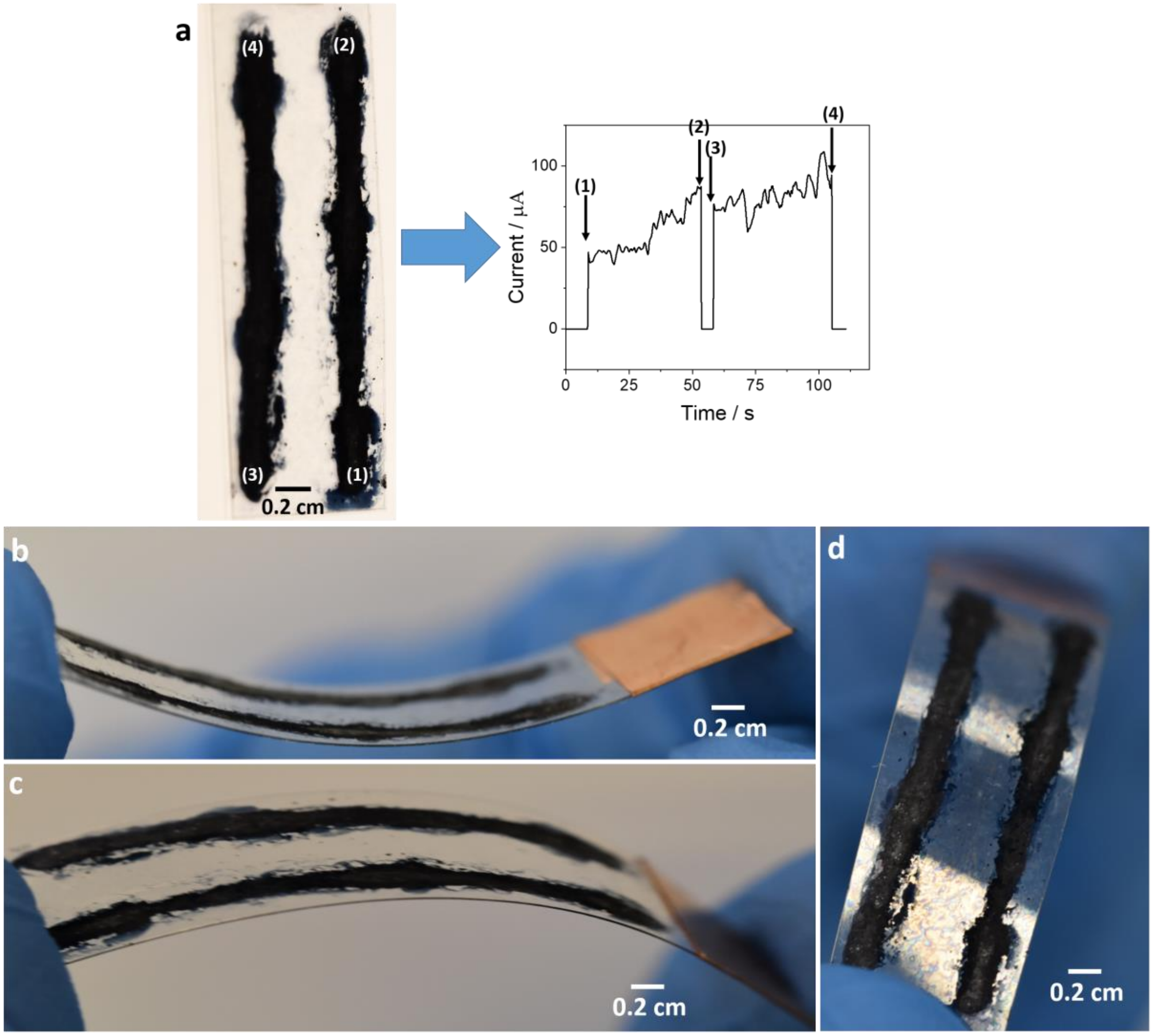


**Supplementary Figure 6 | PEDOT/alginate hydrogel patterning. a** Picture of the two parallel lines of PEDOT/alginate hydrogel (left) and the chronoamperogram of the electrodeposition process (right). The numbers describe the movement of the modified 3D-printer nozzle trough the ITO/PET surface in direct writing. **b-d** The PEDOT/alginate hydrogel under concave, convex and top view of the convex bending.

# References


1. Carioli, G. M. and S. *Tables of Standard Electrode Potentials. John Wiley and Sons Ltd.* vol. 82 (1978).
2. Bard, A. J., Parsons, R. & Jordan, J. *Standard Potentials in Aqueous Solution*. (Routledge, 2017). doi:10.1201/9780203738764.
3. Scialdone, O., Randazzo, S., Galia, A. & Silvestri, G. Electrochemical oxidation of organics in water: Role of operative parameters in the absence and in the presence of NaCl. *Water Res.* **43**, 2260–2272 (2009).
4. Chiang, L.-C., Chang, J.-E. & Wen, T.-C. Indirect oxidation effect in electrochemical oxidation treatment of landfill leachate. *Water Res.* **29**, 671–678 (1995).
5. Gallego, J. H., Castellano, C. E., Calandra, A. J. & Arvia, A. J. The electrochemistry of gold in acid aqueous solutions containing chloride ions. *J. Electroanal. Chem. Interfacial Electrochem.* **66**, 207–230 (1975).
6. Ye, S., Ishibashi, C., Shimazu, K. & Uosaki, K. An In Situ Electrochemical Quartz Crystal Microbalance Study of the Dissolution Process of a Gold Electrode in Perchloric Acid Solution Containing Chloride Ion. *J. Electrochem. Soc.* **145**, 1614–1623 (1998).
7. da Silva, A., Minadeo, M. & de Torresi, S. Gold Nanoparticles and [PEDOT-Poly(D,L-Lactic Acid)] Composite: Synthesis, Characterization and Application to H2O2 Sensing. *J. Braz. Chem. Soc.* (2019) doi:10.21577/0103-5053.20190063.
8. Pestovsky, Y. & Martínez-Antonio, A. Synthesis of gold nanoparticles by tetrachloroaurate reduction with cyclodextrins. *Quim. Nova* (2018) doi:10.21577/0100-4042.20170244.
9. Augusto, T. *et al.* Electrophoretic deposition of Au@PEDOT nanoparticles towards the construction of high-performance electrochromic electrodes. *Sol. Energy Mater. Sol. Cells* **118**, 72–80 (2013).
