# Supplementary material for: Electro-assisted printing of soft hydrogels via controlled electrochemical reactions
Source: Nat Commun. 2022 Mar 15;13:1353. doi: 10.1038/s41467-022-29037-6 (PMC8924165; doi:10.1038/s41467-022-29037-6)
Supplement: Supplementary file 3 — Description of Additional Supplementary Files [file 41467_2022_29037_MOESM3_ESM.pdf]

Description of Additional Supplementary Files

File Name: Supplementary Movie 1
Description: Electrodeposition of the hybrid PEDOT/alginate
